# Pilot study evaluating a brief mindfulness intervention for those with chronic pain: study protocol for a randomized controlled trial

**DOI:** 10.1186/s13063-016-1405-2

**Published:** 2016-06-02

**Authors:** Ana Howarth, Linda Perkins-Porras, Jared G. Smith, Jeevakan Subramaniam, Claire Copland, Mike Hurley, Iain Beith, Muhammad Riaz, Michael Ussher

**Affiliations:** Population Health Research Institute, St George’s, University of London, London, UK; Institute of Medical and Biomedical Education, St George’s, University of London, London, UK; Chronic Pain Service, St George’s University Hospitals NHS Foundation Trust, University of London, London, UK; Faculty of Health, Social Care and Education, Kingston University and St George’s, University of London, London, UK

**Keywords:** Chronic pain, Mindfulness, Intervention, Randomized controlled trial

## Abstract

**Background:**

The burden of chronic pain is a major challenge, impacting the quality of life of patients. Intensive programmes of mindfulness-based therapy can help patients to cope with chronic pain but can be time consuming and require a trained specialist to implement. The self-management model of care is now integral to the care of patients with chronic pain; home-based interventions can be very acceptable, making a compelling argument for investigating brief, self-management interventions. The aim of this study is two-fold: to assess the immediate effects of a brief self-help mindfulness intervention for coping with chronic pain and to assess the feasibility of conducting a definitive randomized controlled trial to determine the effectiveness of such an intervention.

**Methods/Design:**

A randomized controlled pilot study will be conducted to evaluate a brief mindfulness intervention for those with chronic pain. Ninety chronic pain patients who attend hospital outpatient clinics will be recruited and allocated randomly to either the control or treatment group on a 1:1 basis using the computer-generated list of random numbers. The treatment group receives mindfulness audios and the control group receives audios of readings from a non-fiction book, all of which are 15 minutes in length. Immediate effects of the intervention are assessed with brief psychological measures immediately before and after audio use. Mindfulness, mood, health-related quality of life, pain catastrophizing and experience of the intervention are assessed with standardized measures, brief ratings and brief telephone follow-ups, at baseline and after one week and one month. Feasibility is assessed by estimation of effect sizes for outcomes, patient adherence and experience, and appraisal of resource allocation in provision of the intervention.

**Discussion:**

This trial will assess whether a brief mindfulness-based intervention is effective for immediately reducing perceived distress and pain with the side effect of increasing relaxation in chronic pain patients and will determine the feasibility of conducting a definitive randomized controlled trial. Patient recruitment began in January 2015 and is due to be completed in June 2016.

**Trial registration:**

ISRCTN61538090 Registered 20 April 2015

**Electronic supplementary material:**

The online version of this article (doi:10.1186/s13063-016-1405-2) contains supplementary material, which is available to authorized users.

## Background

The burden of chronic pain is a major challenge within healthcare, as approximately 14 million people in England alone currently live with chronic pain [1]. Chronic pain has a negative impact on quality of life [2] and results in high levels of disability [3], with 41 % of patients attending pain clinics reporting being unable to work [4]. Furthermore, high comorbidity rates of depression and anxiety [5] are common, with the 2008 Chief Medical Report stating that 16 % of sufferers report that their chronic pain is so bad that they sometimes want to die [6]. In the UK, the cost for back pain alone has been estimated at £12 billion per annum, which may be partially explained by the fact that, as is reported [6], 25 % of pain sufferers lose their jobs.

Psychological therapies, most commonly in the form of cognitive behavioural therapies [7, 8], have been shown to play an important role in helping patients cope with chronic pain [9, 10]. More recently, mindfulness-based approaches have emerged [11], involving training patients to engage in self-regulation of attention through increasing awareness of, and accepting, present thoughts, feelings and physical sensations [12].

Among those with chronic pain, mindfulness interventions have been shown to reduce anxiety, depression and distress, and to enhance quality of life [13]. Recent UK National Health Service (NHS) guidelines include a recommendation for mindfulness meditation in treating depression [14]. There is also evidence that regular mindfulness meditation modulates neural mechanisms [15, 16], especially those related to pain, as well as benefits for inflammatory systems [17].

While this research is promising, a major barrier with the implementation of current mindfulness interventions is the amount of time they require and the necessity of a trained specialist to oversee them [18]. Mindfulness programmes typically involve weekly group-based sessions over 8 weeks and many chronic pain patients do not have the resources, physically or mentally, to engage with such an intensive programme [19, 20]. Self-help type interventions, which offer more autonomy, are likely to be more adaptable for many such patients and the self-management model of care is now an integral part of the NHS [21]. This model has led to the development of a breadth of interventions with some evidence for efficacy ranging from web-based interventions to brief interventions that patients can readily use as part of a self-care programme.

One type of brief intervention that fits this profile is a short mindfulness-based body or breathing scan. These scans are a key component of mindfulness meditation practice; they involve being directed to focus attention on the present moment through observing the breath and bodily sensations, while becoming aware of, and accepting without judgement, any thoughts and feelings which arise. The traditional mindfulness-based stress reduction intervention includes a body scan [22], lasting anything from 5 to 45 minutes.

There has been little research testing brief mindfulness interventions in either clinical or non-clinical populations. Investigations with healthy populations with a brief mindfulness intervention have been successful in demonstrating a reduction in some aspects of the pain experience, such as distress and sensitivity, during experimental pain studies [23, 24]. An audio-guided 10 minute mindfulness body scan has also been shown to reduce tobacco cravings among abstinent smokers [25, 26]. In a chronic pain population, encouraging effects were found, with an audio recording of a 10-minute body scan reducing reports of distress immediately after listening to the audio in a clinical setting [27]. The intervention evaluated in the current study is a refinement of the intervention used in the latter.

### Aims and objectives

The aim of this paper is to describe the protocol for a pilot randomized controlled trial designed with two primary objectives: assessment of the immediate effects of a brief mindfulness-based intervention on patients with chronic pain and evaluation of the intervention to assess the feasibility of conducting a definitive randomized controlled trial. As regards to the immediate effects of the intervention, it is hypothesized that the intervention will result in a significant reduction in ratings of pain-related symptoms and distress.

## Methods/Design

### Study design

**Fig. 1:**
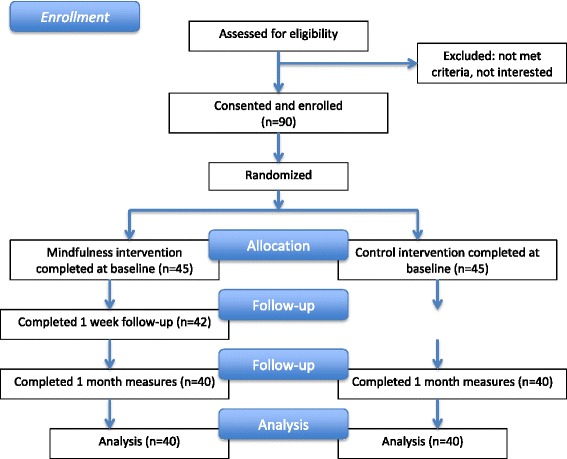
Flow diagram of the feasibility study design assessing the effects of a brief mindfulness intervention for those living with chronic pain

This is a single centre, parallel group, randomized controlled pilot study (see Fig. 1 for a flowchart), designed to assess the immediate effects of a mindfulness-based intervention, as well as the feasibility of conducting a definitive randomized controlled trial.

### Participants and setting

Patients are being recruited from three outpatient NHS physiotherapy/pain clinics at St. George's University Hospitals NHS Foundation Trust. All patients are initially screened by the appropriate clinician (i.e. physiotherapist or pain consultant). Those who meet the inclusion criteria are given a patient information sheet by the clinician and are asked whether they consent to have their contact details passed to a researcher who will call them to discuss whether they wish to join the study or if they would like to meet with the researcher in person to discuss the study.

Patients are eligible if they meet the following criteria: (1) over 18 years of age; (2) living with chronic pain (i.e. those who live with a diagnosis of chronic pain or those who have had pain for more than three months after the time healing should have occurred [19]); and (3) able to hear audio recordings or have their own equipment to enable them to do so.

Patients are excluded if they are (1) considered by the clinician to be too unwell to participate or (2) unable to speak or read English sufficiently to understand and complete the self-administered questionnaires.

### Sample size

It is recommended that pilot/feasibility studies ideally recruit a total of at least 50 participants [20], although in practice many studies recruit 50–100 participants. We aim to recruit 90 participants (45 in each treatment arm). Then, allowing for 10 participants withdrawing (estimate based on a previous mindfulness study with the same population [27]), we aim to have around 80 participants with data through to the final one month follow-up. Moreover, based on a finding from a previous similar study [27], we used a Wilcoxon signed-rank test (G-Power software) to calculate that a total sample size of at least 50 participants would be required to detect a significant difference of 1.2 (SD = 2) on the perceived distress scale, between the two groups (immediately) after the intervention. This was with 80 % power at the 5 % significance level.

### Randomization

An independent statistician generates a randomization list using the online resource ‘Research Randomizer’. This list is used by researchers to allocate volunteers to either the control or treatment group on a 1:1 basis. Patients are allocated their number in ascending order based on their order of enrolment. Allocation is concealed from the participant and researcher until all baseline assessments have been completed. Due to limited resources, the same researcher delivers the intervention and administers the research measures, and therefore treatment allocation cannot be concealed from the researcher. Thus, neither participants nor researchers are blinded to treatment allocation during intervention delivery or during outcome assessment. An independent statistician, who is blinded to the treatment allocation, will complete the initial analysis for the main outcomes.

### Interventions

To improve the reporting of the interventions, the Template for Intervention Description and Replication [28] and the SPIRIT (Standard Protocol Items: Recommendations for Interventional Trials) checklist and figure [29] have been used to guide the description of the interventions. The SPIRIT checklist itself is attached in Additional file 1, presenting items addressed in the reporting of the protocol overall and the SPIRIT figure is attached in Additional file 2, summarizing a schedule of enrolment, interventions, and assessments.

#### Treatment group: brief self-management mindfulness-based audios

Patients in the treatment group are given an audio recording of a 15-minute mindfulness body scan on an MP3 player (with earphones) or are offered the option of having the audio downloaded directly to a personal device of their choice, such as a smart phone or iPad.

The choice of the body scan meditation for the audio was based on successful traditional mindfulness-based stress reduction interventions, which routinely include a body scan meditation as the introductory exercise. In a clinical setting with a chronic pain population, a brief (10 minute) version of the body scan was found to reduce reports of distress, immediately after listening to the audio [27].

The body scan used in this study is an extended version of a 10-minute body scan used in a qualitative study (to be published elsewhere) investigating the acceptability of the intervention to patients. It is based on a transcript from Breathworks [30], an established mindfulness organisation specialising in supporting those with chronic pain. As part of the prior qualitative study, and in response to feedback from patients, the intervention was extended from 10 to 15 minutes so that it would feel less rushed.

The audio recording directs the listener to systematically ‘scan’ their body with their attention, starting with the toes and finishing with the crown of the head. Throughout this process, the listener is also encouraged to be aware of their breathing and to accept all thoughts and feelings, whether positive or negative, without trying to alter them in any way. This intervention is administered face-to-face for the initial use by a researcher in a clinical setting (i.e. physiotherapy/medical side room or cubicle) and telephone follow-up at one week and one month is conducted by the same researcher. Use of the audio at least three further times during the first week is requested and, after that, use is encouraged but no set number of times is prescribed for the following three weeks. Following administration of the intervention, a study packet, including information and instructions for use of the audios along with brief information regarding mindfulness (i.e. frequently asked questions) and questionnaires to be filled out at home, is given to the patient. The inclusion of an information sheet was developed in response to patient feedback in the previous qualitative study.

An audio of a breathing meditation and a moving meditation are given to the treatment group as well, but use is not recommended until after one week. The breathing meditation is an exercise where breath is used as an object of concentration and the listener is asked to focus on the sensations of breathing (e.g. the feeling of the chest rising and falling). The moving meditation is focused on gentle exercises (e.g. small wrist twists or arm movements), which can be done sitting or standing and the listener is guided to pay attention to bodily sensations after making each movement. Both are based on transcripts from Breathworks.

#### Control group: relaxation/distraction audios

Patients in the control group are given eight, 15-minute audio recordings of sequential readings from “The English Village: History and Traditions” [31], which is a non-fiction book considered to be neutral in nature. The readings start from the beginning of the book and the hope is that enough interest is generated as the story progresses, so as to encourage patients to listen to a total of four sessions in the first week as easily as those in the treatment group. In total, eight sessions were recorded with the intention that four recordings would be used in the first week and that the remaining four could be used in the following three weeks. As with the treatment group, patients are given an MP3 player (with earphones) or the option of having the audios downloaded directly to a personal device. For the first session in clinic, the first of these sequential readings, which is also the first section of the book, is presented. Non-fiction material, similar in style and content, has been used in a previous study examining the acute effects of mindfulness among those with chronic pain, where it has been found to be an acceptable intervention [27]. Recordings were made using the same narrator as the intervention, and are read at a similar pace and with comparable pauses.

As with the treatment group, use of the audios is requested at least three further times during the first week. After that, continuing use is encouraged, with no set prescription for the following three weeks. Following administration of the control intervention, the study packet, including information and instructions for use of the audios (minus the mindfulness frequently asked questions that are included for the treatment group) and questionnaires to be filled out at home, is given to the patient.

### Procedure in clinic

Patients who meet the inclusion criteria are approached by the research team and given a patient information sheet regarding the study. Patients are given as much time as they want to consider whether they want to participate and written consent is obtained from all patients who agree to do so. Delivery of the intervention is standardized, with a researcher checklist (i.e. written manual) including a step-by-step script developed to address fidelity issues. This script and checklist are used during recruitment and researchers observed each other administering the intervention to at least one patient each so as to standardize procedures.

As detailed previously, an initial face-to-face visit is conducted in a private room or cubicle at St George’s Hospital or St John’s Therapy Centre. Patients are randomized to either the control or treatment group and asked to complete baseline measures, including brief psychological measures, and to listen to the relevant audio once in clinic with the researcher. After listening to the audio, patients are asked to complete the brief psychological measures again. Before leaving, patients are advised to consider barriers and solutions to use of the audio in their own environment and are given a study packet to take home. They are instructed to use the audios as a self-management tool and to try the audio during particularly painful times if possible. The contents of the study packet containing follow-up questionnaires (i.e. Study Diaries 1–3), self-addressed prepaid return envelopes and brief instructions, are then reviewed with the patient in case there are any queries. If the audios are not directly downloaded to a personal device, patients are allowed to keep the MP3 players if they want after they finish, as they are a minimal cost. The offer of the MP3 player is not mentioned in the patient information sheet, and therefore is not considered as an incentive to recruitment.

### Measures and schedule of assessment

#### Baseline data collection

Patients are asked to provide demographic details including age, marital status, occupation, education and ethnic group along with five pain-related questions, namely: “What is your clinical diagnosis?”, “How long have you been living with your pain?”, “Are you currently taking any medication for your pain and if so, which one/s?”, “Over the last week, how confident have you been in managing your pain” (1 = not at all confident to 7 = extremely confident), and “During the past week, how much has your work or other regular daily activities been limited as a result of your pain symptoms?” (1 = not at all to 5 = extremely). They then complete a measure of mood (Hospital Anxiety and Depression Scale; HADS [32]), a mindfulness questionnaire (Cognitive and Affective Mindfulness Scale-Revised; CAMS-R [33]), a pain-specific questionnaire (Brief Pain Inventory; BPI [34]), a pain catastrophizing questionnaire (Pain Catastrophizing Scale; PCS [35]), and a health-related quality of life (HRQoL) questionnaire (EuroQol – 5 Dimension – 5 Levels; EQ-5D-5 L [36]). Immediately before and after the initial use of the audio in clinic, patients are asked to complete three questions regarding their level of distraction, pain severity and pain distress (1 = not at all to 5 = extremely). Full details of the measures are given below.

#### Measures to be completed during the first week

Study Diary 1 includes a self-monitoring table detailing date, time and position of use (e.g. sitting or lying) of the audios and a repeat of the baseline brief measures of level of distraction, pain severity and pain distress immediately before and after the last session of listening to the audio during the first week.

#### Measures to be completed after one week

Study Diary 2 includes a brief questionnaire where patients are asked: “How useful did you find the audio guide for helping you to relax?” (1 = not at all to 5 = extremely useful), and “Would you recommend this audio guide to others to help manage their chronic pain?” (1 = definitely would not recommend to 5 = definitely would recommend it). To assess level of experience of activities related to mindfulness, the question: “Have you had experience of yoga, tai-chi or any type of meditation?” (1 = no experience of these activities to 7 = I currently practice these activities at least once a week) is included. These questions are followed by a repeat of the measure of mindfulness that was completed at baseline so as to detect any early changes in mindfulness present after one week.

#### Measures to be completed during and after one month

Study Diary 3 includes another self-monitoring table where patients can continue to detail date, time and position of use of the audios during the three weeks prior to the final 1 month follow-up. At 1 month, items regarding pain self-efficacy and physical function are repeated in addition to the measures of mood, pain catastrophizing, mindfulness, and HRQoL that were administered at baseline. While one week is likely too brief for patients to make (meaningful) changes in physical and/or psychological function, assessment after the completion of the intervention (one month) will better allow for the detection of changes in function where they have occurred.

A brief assessment of whether they have continued listening to the audio (and if so, how often), a discussion of the main barriers to and facilitators of use, and their view on the beneficiality of an online support group forum, texting support and more face time, is conducted with a brief (around 5 minutes) open-ended telephone interview. A schedule of assessment for all measures included is presented in Table 1 below.

**Table 1:** Schedule of data and measurement collection

Measure	Baseline	During week	At 1 week	During month	At 1 month
Background and pain-related questionnaire	X				
Pain self-efficacy item	X				X
Pain and physical function item	X				X
Mood questionnaire (HADS)	X				
Mindfulness questionnaire (CAMS-R)	X		X		X
Pain-specific questionnaire (BPI)	X				
Pain catastrophizing questionnaire (PCS)	X				X
Health-related quality of life questionnaire (EQ-5D-5L)	X				X
Brief psychological measures (two times, before and after intervention)	X	X			
Experience of audio items			X		
Previous experience			X		
Self-monitoring table		X		X	

HADS, Hospital Anxiety and Depression Scale; CAMS-R, Cognitive and Affective Mindfulness Scale Revised; BPI, Brief Pain Inventory; PCS, Pain Catastrophizing Scale; EQ-5D-5 L, EuroQuol – 5 Dimensions – 5 Levels

### Intervention behaviour change techniques at one week

At one week, the researcher follows up by telephone and encourages continued use of the intervention, identifies perceived barriers and benefits of use and sets goals with the patient by recommending continued use of the intervention at least three times a week. Self-monitoring by diary is also encouraged. These behaviour change techniques come under the labels “Goal setting” or “Action planning”, “Self-monitoring of behaviour” and “Problem solving” as per the Behavior Change Techniques Taxonomy (v1) [37], which is the established standardized taxonomy for behaviour change techniques.

### Debrief at one month

Patients are followed up after one month by telephone for a study debrief. Patients are debriefed regarding the full nature of the study and, if they are part of the control group, they are offered to have the intervention audios sent to them in case they wish to try them. Resources that are readily available to the public are recommended at this time if patients wish to further explore mindfulness. Patients are reminded to complete and post back the questionnaires.

### Measures

#### Hospital Anxiety and Depression Scale

The HADS is an easily-administered screening questionnaire designed by Zigmond and Snaith [32], which has been widely used as a tool to assess the severity of depression and anxiety. Patients are asked to respond to 14 items, seven measuring anxiety and seven measuring depression. The respondent must choose one of four responses for each item according to how they have felt over the previous week. A score of 0–21 is calculated for each disorder with total scores between 11–21 indicating abnormal levels of anxiety/depression [38]. The HADS has also been routinely used for research within chronic pain populations [39–42].

#### Cognitive and Affective Mindfulness Scale–Revised

The CAMS-R [33] is a revised version of the Cognitive and Affective Mindfulness Scale [43], which is an 18-item measure designed to capture mindfulness as a general daily experience. The CAMS-R is a 10-item scale which uses everyday language appropriate for those with little meditation experience and has been compared with two other existing mindfulness measures, the Mindfulness Attention Awareness Scale [44] and The Freiburg Mindfulness Inventory [45], where it was found to be positively correlated (r = 0.51, *P* < 0.001 and r = 0.66, *P* < 0.001, respectively) [46, 47], with an acceptable internal consistency (a = 0.76) [33], which was a weakness of the original scale. The CAMS-R is unique in that it is related to psychological distress, which is highly relevant to the current study and chronic pain population.

#### EuroQuol – 5 Dimensions – 5 Levels

The EQ-5D-5 L [36] is the most recently developed version of the EQ-5 Dimensions [48, 49], which is a standardised measure of health status that has good construct validity and responsiveness among people with chronic pain [50]. Developed by the EuroQol group, it is supported by the National Institute for Clinical Excellence (NICE) for measuring change in health-related quality of life with various patient groups [51], has been validated within numerous patient groups including the chronic pain population, and has been shown to be a sensitive tool with internal consistency and reliability [52–55].

#### The Brief Pain Inventory

The BPI [34] is an easy to use tool for the assessment of pain in both clinical and research settings and uses simple numeric rating scales from 0 to 10 (with 0 = no pain to 10 = pain as bad as you can imagine). The BPI has been used internationally [34, 56, 57] to measure severity and interference of pain in patients who live with a range of chronic pain presentations.

#### Pain Catastrophizing Scale

The PCS [35] is a 13-item scale consisting of statements regarding the thoughts and feelings that patients report when they experience pain scored from 0 (not at all) to 4 (all the time). Total PCS scores range from 0 to 52 points and higher scores indicating higher levels of pain catastrophizing. The PCS consists of three subscales, which are magnification, rumination, and helplessness. The scale was developed to be used within both clinical and non-clinical populations and was originally an elaboration on the Coping strategies Questionnaire [58]. The PSC has been shown to have reliability and validity in both pain populations and healthy adult populations with a high internal consistency [59].

#### Brief measures to be completed before and after audio in clinic and the last session during the first week at home

Three brief, single-item measures are used to assess level of distractedness, pain severity and pain distress. Patients are asked to rate “How distracted do you feel right now?” (devised specifically for this study), “How severe are your pain related symptoms right now?”, and “How distressing are your pain related symptoms right now?”, all on a scale from 1 (not at all) to 7 (extremely so).

### Statistical analysis

We will present the baseline characteristics according to the two study groups. For the analysis assessing the immediate effects of the intervention, we will assess the effect of the body scan versus the control intervention on ratings for the brief psychological measures administered immediately before and after the interventions. This analysis will be conducted for the ‘before’ and ‘after’ ratings in the clinic and also for those conducted in the participant’s own environment. First, we will compute change scores by subtracting post-intervention scores from pre-intervention scores. As it is a pilot study with a small sample size, initially we will assess the effect of the treatment groups on outcomes (i.e. change scores) using *t* tests or Mann–Whitney tests. We will then conduct multiple linear regressions with the change scores as the dependent variables and treatment groups as the independent variable. Statistical significance will be assessed with the likelihood-ratio test, with the estimate of effect given as mean difference of change scores and 95 % confidence interval. As a secondary analysis we will adjust for age, sex and baseline BPI score, as potentially important prognostic baseline factors. We will also inspect the baseline characteristics, for the two groups, to assess whether there are any other potential confounders that need to be controlled for.

Next, we will assess the effect of the study groups on outcomes recorded at baseline and one month. All participants with data at both time points will be included in the analysis. It is unlikely that the study will be sufficiently powered to detect significant differences between the groups but we will carry out analyses in order to inform parameters for a definitive trial. We will compute change scores between baseline and one month for the HADS, EQ-5D-5 L, PCS and the CAMS-R, and we will conduct multiple regressions, with adjustments as above. We will also repeat this procedure for the CAMS-R at baseline and one week.

Before conducting any of the regression analyses, we will assess the distribution of residuals of the dependent variable(s). We will use the bootstrap method if the distribution of the residuals of the dependent variable in any regression model is not normal. In the case of missing data at one week or one month, we will conduct a sensitivity analysis, in which we will use multiple imputation to impute values for those with missing data for any variables.

We will use *t* tests or Mann–Whitney tests to compare scores for ratings of ‘usefulness’ and for whether participants would recommend the intervention. All data will be analysed using SPSS V19, with the level of significance set at *P* < 0.05.

## Discussion

As consistent, accessible chronic pain management is a huge challenge across the NHS, testing the feasibility of a brief, accessible intervention that could be easily introduced into current NHS practice addresses a key issue in the area of chronic pain research. The usefulness and logistics of implementing a brief self-management intervention into the existing NHS environment is assessed with this study, which has been designed to investigate both acute effects and feasibility. The acute effects will provide an indication of how effective the intervention is and the feasibility assessment will inform the potential likelihood of conducting a definitive randomized controlled trial investigating effectiveness and cost effectiveness of a brief self-help mindfulness tool. Eligibility, recruitment and retention rates will be audited along with resources used in provision of the intervention, including costs of the MP3 players, telephone calls, text messages and staff time needed for intervention delivery. This information alongside patients’ adherence to the treatment regimen, experiences of the intervention, and its acceptability and usefulness will allow for a rigorous and well-designed definitive randomized controlled trial to be conducted if findings are positive.

One of the main limitations of the study is that, for pragmatic reasons, the researcher assessing outcomes is not blinded to the participants’ intervention status. We have attempted to minimize researcher bias by, as far as possible, assessing patient outcomes with validated self-report instruments. Additionally, to further minimize bias, the statistician analysing the data will be blind to the treatment allocation of the participants. It is hoped that a definitive trial would take measures to ensure that the researchers assessing outcomes are blinded to the allocation of participants. Another noteworthy limitation is our inability to monitor patient adherence to the intervention when listening to the audio at home. It is anticipated that, in a full trial, ecological momentary assessment would be used to monitor adherence, including having an audio player that records the level of usage.

### Current study status

Patient recruitment began in January 2015 and is due to be completed in July 2016.

## Abbreviations

BPI, Brief Pain Inventory; CAMS-R, Cognitive and Affective Mindfulness Scale-Revised; EQ-5D-5 L, EuroQol – 5 Dimension – 5 Levels; HADS, Hospital Anxiety and Depression Scale; NHS, National Health Service; PCS, Pain Catastrophizing Scale

## Additional files

Additional file 1: SPIRIT checklist. (DOC 744 kb) Additional file 2: SPIRIT figure. (DOC 52 kb)

## References

[1] Department of Health. Long Term Conditions Compendium of Information. Third ed. 2012. https://www.gov.uk/government/publications/long-term-conditions-compendium-of-informationthird-edition.

[2] Bridges S. Health Survey for England 2011: Chronic pain. 2012. Chapter 9. pp. 291–32. http://www.hscic.gov.uk/catalogue/PUB09300/HSE2011-Ch9-Chronic-Pain.pdf.

[3] Fredheim OMS, Kaasa S, Fayers P, Saltnes T, Jordhøy M, Borchgrevink PC (2008). Chronic non-malignant pain patients report as poor health-related quality of life as palliative cancer patients. Acta Anaesthesiol Scand.

[4] British Pain Society (2012). National Pain Audit Final Report 2010–2012.

[5] Elliott T, Renier C, Palcher J (2003). Chronic pain, depression, and quality of life: correlations and predictive value of the SF-36. Pain Med..

[6] Sir Liam Donaldson CMO (2008). 150 years of the Annual Report of the Chief Medical Officer: On the state of public health.

[7] Morley S, Eccleston C, Williams A (1999). Systematic review and meta-analysis of randomized controlled trials of cognitive behaviour therapy and behaviour therapy for chronic pain in adults, excluding headache. Pain.

[8] Eccleston C, Williams AC, Morley S (2009). Psychological therapies for the management of chronic pain (excluding headache) in adults. Cochrane Database Syst Rev..

[9] Williams AC, Eccleston C, Morley S (2012). Psychological therapies for the management of chronic pain (excluding headache) in adults. Cochrane Database Syst Rev..

[10] Roditi D, Robinson ME (2011). The role of psychological interventions in the management of patients with chronic pain. Psychol Res Behav Manag..

[11] Hayes SC (2004). Acceptance and commitment therapy, relational frame theory, and the third wave of behavioral and cognitive therapies. Behav Ther..

[12] Kabat-Zinn J (1990). Full catastrophe living: using the wisdom of your body and mind to face stress, pain, and illness.

[13] Hofmann SG, Sawyer AT, Witt AA, Oh D (2010). The effect of mindfulness-based therapy on anxiety and depression: a meta-analytic review. J Consult Clin Psychol.

[14] National Collaborating Centre for Mental Health (2010). Depression: the treatment and management of depression in adults. NICE Clinical Guidelines, No. 90.

[15] Zeidan F, Martucci KT, Kraft RA, Gordon NS, McHaffie JG, Coghill RC (2011). Brain mechanisms supporting the modulation of pain by mindfulness meditation. J Neurosci.

[16] Zeidan F, Grant JA, Brown CA, McHaffie JG, Coghill RC (2012). Mindfulness meditation-related pain relief: evidence for unique brain mechanisms in the regulation of pain. Neurosci Lett.

[17] Greeson JM (2008). Mindfulness Research Update: 2008. Complement Health Pract Rev..

[18] World Health Organization (2003). Adherence to long-term therapies: Evidence for action.

[19] The British Pain Society – FAQs. 2008. https://www.britishpainsociety.org/people-with-pain/frequently-asked-questions/. Accessed 15 Sept 2015.

[20] Sim J, Lewis M (2012). The size of a pilot study for a clinical trial should be calculated in relation to considerations of precision and efficiency. J Clin Epidemiol.

[21] Rogers A, Kennedy A, Bower P, Gardner C, Gately C, Lee V, Reeves D, Richardson G (2008). The United Kingdom Expert Patients Programme: results and implications from a national evaluation. Med J Aust.

[22] Baer R (2003). Mindfulness training as a clinical intervention: a conceptual and empirical review. Clin Psychol Sci Pract.

[23] Zeidan F, Gordon NS, Merchant J, Goolkasian P (2010). The effects of brief mindfulness meditation training on experimentally induced pain. J Pain..

[24] Liu WS, Wang S, Liu X, Chang S, Chen W, Si M (2013). Effect of brief mindfulness intervention on tolerance and distress of pain induced by cold-pressor task. Stress Heal..

[25] Cropley M, Ussher M, Charitou E (2007). Acute effects of a guided relaxation routine (body scan) on tobacco withdrawal symptoms and cravings in abstinent smokers. Addiction.

[26] Ussher M, Cropley M, Playle S, Mohidin R, West R (2009). Effect of isometric exercise and body scanning on cigarette cravings and withdrawal symptoms. Addiction.

[27] Ussher M, Spatz A, Copland C, Nicolaou A, Cargill A, Amini-Tabrizi N (2014). Immediate effects of a brief mindfulness-based body scan on patients with chronic pain. J Behav Med.

[28] Hoffmann TC, Glasziou PP, Boutron I, Milne R, Perera R, Moher D (2014). Better reporting of interventions: template for intervention description and replication (TIDieR) checklist and guide. BMJ..

[29] Chan A-W, Tetzlaff JM, Altman DG, Laupacis A, Gøtzsche PC, Krleža-Jerić K (2013). SPIRIT 2013 statement: defining standard protocol items for clinical trials. Ann Intern Med.

[30] Breathworks Mindfulness. http://www.breathworks-mindfulness.org.uk/. Accessed 15 Sept 2015.

[31] Wainwright M (2011). The English Village: History and Traditions.

[32] Zigmond AS, Snaith RP (1983). The hospital anxiety and depression scale. Acta Psychiatr Scand.

[33] Feldman G, Hayes A, Kumar S, Greeson J, Laurenceau J (2007). Mindfulness and emotion regulation: the development and initial validation of the cognitive and affective mindfulness scale-revised (CAMS-R). J Psychopathol Behav Assess.

[34] Cleeland CS, Ryan KM (1994). Pain assessment: global use of the Brief Pain Inventory. Ann Acad Med Singapore.

[35] Sullivan MJL, Bishop SR, Pivik J (1995). The Pain Catastrophizing Scale: development and validation. Psychol Assess.

[36] Herdman M, Gudex C, Lloyd A, Janssen M, Kind P, Parkin D (2011). Development and preliminary testing of the new five-level version of EQ-5D (EQ-5D-5 L). Qual Life Res.

[37] Michie S, Richardson M, Marie J, Abraham C, Frances J, Hardemann W (2013). The behavior change technique taxonomy (v1) of 93 hierarchically clustered techniques: building an international consensus for the reporting of behavior change interventions. Ann Behav Med..

[38] Crawford JR, Henry JD, Crombie C, Taylor EP (2001). Normative data for the HADS from a large non-clinical sample. Br J Clin Psychol.

[39] Sagheer MA, Khan MF, Sharif S (2013). Association between chronic low back pain, anxiety and depression in patients at a tertiary care centre. J Pak Med Assoc.

[40] Tang NKY, Wright KJ, Salkovskis PM (2007). Prevalence and correlates of clinical insomnia co-occurring with chronic back pain. J Sleep Res.

[41] Kalia LV, O’Connor PW (2005). Severity of chronic pain and its relationship to quality of life in multiple sclerosis. Mult Scler.

[42] Veehof MM, Oskam M-J, Schreurs KMG, Bohlmeijer ET (2011). Acceptance-based interventions for the treatment of chronic pain: a systematic review and meta-analysis. Pain.

[43] Feldman G, Hayes A (2005). Preparing for problems: a measure of mental anticipatory processes. J Res Pers.

[44] Brown KW, Ryan RM (2003). The benefits of being present: mindfulness and its role in psychological well-being. J Pers Soc Psychol.

[45] Walach H, Buchheld N, Buttenmüller V, Kleinknecht N, Schmidt S (2006). Measuring mindfulness—the Freiburg Mindfulness Inventory (FMI). Pers Individ Dif.

[46] Thompson BL, Waltz J (2007). Everyday mindfulness and mindfulness meditation: overlapping constructs or not?. Pers Individ Dif.

[47] Baer RA, Smith GT, Hopkins J, Krietemeyer J, Toney L (2006). Using self-report assessment methods to explore facets of mindfulness. Assessment.

[48] Brooks R (1996). EuroQol: the current state of play. Health Policy.

[49] EuroQol Group (1990). EuroQol--a new facility for the measurement of health-related quality of life. Health Policy.

[50] Obradovic M, Lal A, Liedgens H (2013). Validity and responsiveness of EuroQol-5 dimension (EQ-5D) versus Short Form-6 dimension (SF-6D) questionnaire in chronic pain. Health Qual Life Outcomes..

[51] Brazier J, Longworth L. NICE DSU technical support document 8: an introduction to the measurement and valuation of health for NICE submissions report by the Decision Support Unit. Sheffield: NICE DSU (Decision Support Unit). 2011. http://www.nicedsu.org.uk/TSD8%20Introduction%20to%20MVH_final.pdf. 28481495

[52] Dorman PJ, Waddell F, Slattery J, Dennis M, Sandercock P (1997). Is the EuroQol a valid measure of health-related quality of life after stroke?. Stroke.

[53] Mustur D, Vesović-Potić V, Stanisavljević D, Ille T, Ille M (2009). Assessment of functional disability and quality of life in patients with ankylosing spondylitis. Srp Arh Celok Lek.

[54] Hurst NP, Jobanputra P, Hunter M, Lambert M, Lochhead A, Brown H (1994). Validity of Euroqol--a generic health status instrument--in patients with rheumatoid arthritis. Economic and Health Outcomes Research Group. Br J Rheumatol.

[55] Marra CA, Woolcott JC, Kopec JA, Shojania K, Offer R, Brazier JE (2005). A comparison of generic, indirect utility measures (the HUI2, HUI3, SF-6D, and the EQ-5D) and disease-specific instruments (the RAQoL and the HAQ) in rheumatoid arthritis. Soc Sci Med.

[56] Gjeilo KH, Stenseth R, Wahba A, Lydersen S, Klepstad P (2007). Validation of the brief pain inventory in patients six months after cardiac surgery. J Pain Symptom Manage.

[57] Song C-Y, Lin S-F, Huang C-Y, Wu H-C, Chen C-H, Hsieh C-L. Validation of the Brief Pain Inventory in patients with low back pain. Spine (Phila Pa 1976). 2016. [EPub ahead of print.] 10.1097/BRS.0000000000001478 26839985

[58] Rosenstiel AK, Keefe FJ (1983). The use of coping strategies in chronic low back pain patients: relationship to patient characteristics and current adjustment. Pain.

[59] Osman A, Barrios FX, Gutierrez PM, Kopper BA, Merrifield T, Grittmann L (2000). The Pain Catastrophizing Scale: further psychometric evaluation with adult samples. J Behav Med.

