# Supplementary material for: Pilot study evaluating a brief mindfulness intervention for those with chronic pain: study protocol for a randomized controlled trial
Source: Trials. 2016 Jun 2;17:273. doi: 10.1186/s13063-016-1405-2 (PMC4890280; doi:10.1186/s13063-016-1405-2)
Supplement: Additional file 1: — SPIRIT checklist. (DOC 744 kb) [file 13063_2016_1405_MOESM1_ESM.doc]

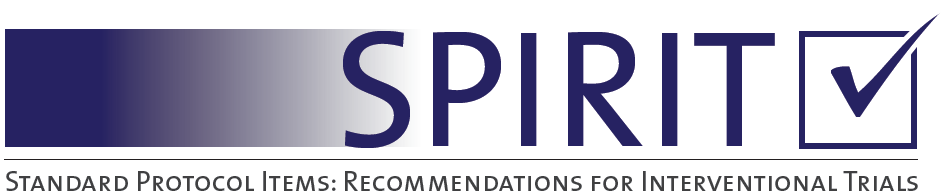


SPIRIT 2013 Checklist: Recommended items to address in a clinical trial protocol and related documents*

| Section/item | Item No | Description | Addressed on page number |
| --- | --- | --- | --- |
| **Administrative information** | | |  |
| Title | 1 | Descriptive title identifying the study design, population, interventions, and, if applicable, trial acronym | 1 |
| Trial registration | 2a | Trial identifier and registry name. If not yet registered, name of intended registry | 3 |
|  | 2b | All items from the World Health Organization Trial Registration Data Set |  |
| Protocol version | 3 | Date and version identifier | 1 |
| Funding | 4 | Sources and types of financial, material, and other support | 21, see Patient Information Sheet (PIS) Appendix 1 |
| Roles and responsibilities | 5a | Names, affiliations, and roles of protocol contributors | 20 |
|  | 5b | Name and contact information for the trial sponsor | 20 |
|  | 5c | Role of study sponsor and funders, if any, in study design; collection, management, analysis, and interpretation of data; writing of the report; and the decision to submit the report for publication, including whether they will have ultimate authority over any of these activities | 20 |
|  | 5d | Composition, roles, and responsibilities of the coordinating centre, steering committee, endpoint adjudication committee, data management team, and other individuals or groups overseeing the trial, if applicable (see Item 21a for data monitoring committee) | 20 |
| Introduction |  |  |  |
| Background and rationale | 6a | Description of research question and justification for undertaking the trial, including summary of relevant studies (published and unpublished) examining benefits and harms for each intervention | 3 |
|  | 6b | Explanation for choice of comparators | 11 |
| Objectives | 7 | Specific objectives or hypotheses | 5 |
| Trial design | 8 | Description of trial design including type of trial (eg, parallel group, crossover, factorial, single group), allocation ratio, and framework (eg, superiority, equivalence, noninferiority, exploratory) | 6 |
| Methods: Participants, interventions, and outcomes | | |  |
| Study setting | 9 | Description of study settings (eg, community clinic, academic hospital) and list of countries where data will be collected. Reference to where list of study sites can be obtained | 6 |
| Eligibility criteria | 10 | Inclusion and exclusion criteria for participants. If applicable, eligibility criteria for study centres and individuals who will perform the interventions (eg, surgeons, psychotherapists) | 7 |
| Interventions | 11a | Interventions for each group with sufficient detail to allow replication, including how and when they will be administered | 8 |
|  | 11b | Criteria for discontinuing or modifying allocated interventions for a given trial participant (eg, drug dose change in response to harms, participant request, or improving/worsening disease) | See PIS |
|  | 11c | Strategies to improve adherence to intervention protocols, and any procedures for monitoring adherence (eg, drug tablet return, laboratory tests) | 12 |
|  | 11d | Relevant concomitant care and interventions that are permitted or prohibited during the trial |  |
| Outcomes | 12 | Primary, secondary, and other outcomes, including the specific measurement variable (eg, systolic blood pressure), analysis metric (eg, change from baseline, final value, time to event), method of aggregation (eg, median, proportion), and time point for each outcome. Explanation of the clinical relevance of chosen efficacy and harm outcomes is strongly recommended | 12 |
| Participant timeline | 13 | Time schedule of enrolment, interventions (including any run-ins and washouts), assessments, and visits for participants. A schematic diagram is highly recommended (see Figure) | 12 |
| Sample size | 14 | Estimated number of participants needed to achieve study objectives and how it was determined, including clinical and statistical assumptions supporting any sample size calculations | 7-8 |
| Recruitment | 15 | Strategies for achieving adequate participant enrolment to reach target sample size | 7 |
| **Methods: Assignment of interventions (for controlled trials)** | | |  |
| Allocation: |  |  |  |
| Sequence generation | 16a | Method of generating the allocation sequence (eg, computer-generated random numbers), and list of any factors for stratification. To reduce predictability of a random sequence, details of any planned restriction (eg, blocking) should be provided in a separate document that is unavailable to those who enrol participants or assign interventions | 8 |
| Allocation concealment mechanism | 16b | Mechanism of implementing the allocation sequence (eg, central telephone; sequentially numbered, opaque, sealed envelopes), describing any steps to conceal the sequence until interventions are assigned | 8 |
| Implementation | 16c | Who will generate the allocation sequence, who will enrol participants, and who will assign participants to interventions | 8 |
| Blinding (masking) | 17a | Who will be blinded after assignment to interventions (eg, trial participants, care providers, outcome assessors, data analysts), and how | 8 |
|  | 17b | If blinded, circumstances under which unblinding is permissible, and procedure for revealing a participant’s allocated intervention during the trial |  |
| **Methods: Data collection, management, and analysis** | | |  |
| Data collection methods | 18a | Plans for assessment and collection of outcome, baseline, and other trial data, including any related processes to promote data quality (eg, duplicate measurements, training of assessors) and a description of study instruments (eg, questionnaires, laboratory tests) along with their reliability and validity, if known. Reference to where data collection forms can be found, if not in the protocol | 11 |
|  | 18b | Plans to promote participant retention and complete follow-up, including list of any outcome data to be collected for participants who discontinue or deviate from intervention protocols | 12 |
| Data management | 19 | Plans for data entry, coding, security, and storage, including any related processes to promote data quality (eg, double data entry; range checks for data values). Reference to where details of data management procedures can be found, if not in the protocol | 6 |
| Statistical methods | 20a | Statistical methods for analysing primary and secondary outcomes. Reference to where other details of the statistical analysis plan can be found, if not in the protocol | 18-19 |
|  | 20b | Methods for any additional analyses (eg, subgroup and adjusted analyses) | 18-19 |
|  | 20c | Definition of analysis population relating to protocol non-adherence (eg, as randomised analysis), and any statistical methods to handle missing data (eg, multiple imputation) | 19 |
| **Methods: Monitoring** | | |  |
| Data monitoring | 21a | Composition of data monitoring committee (DMC); summary of its role and reporting structure; statement of whether it is independent from the sponsor and competing interests; and reference to where further details about its charter can be found, if not in the protocol. Alternatively, an explanation of why a DMC is not needed | P6 and PIS |
|  | 21b | Description of any interim analyses and stopping guidelines, including who will have access to these interim results and make the final decision to terminate the trial |  |
| Harms | 22 | Plans for collecting, assessing, reporting, and managing solicited and spontaneously reported adverse events and other unintended effects of trial interventions or trial conduct |  |
| Auditing | 23 | Frequency and procedures for auditing trial conduct, if any, and whether the process will be independent from investigators and the sponsor | 19 |
| Ethics and dissemination | | |  |
| Research ethics approval | 24 | Plans for seeking research ethics committee/institutional review board (REC/IRB) approval | 6 |
| Protocol amendments | 25 | Plans for communicating important protocol modifications (eg, changes to eligibility criteria, outcomes, analyses) to relevant parties (eg, investigators, REC/IRBs, trial participants, trial registries, journals, regulators) |  |
| Consent or assent | 26a | Who will obtain informed consent or assent from potential trial participants or authorised surrogates, and how (see Item 32) | 6, 11 |
|  | 26b | Additional consent provisions for collection and use of participant data and biological specimens in ancillary studies, if applicable | NA |
| Confidentiality | 27 | How personal information about potential and enrolled participants will be collected, shared, and maintained in order to protect confidentiality before, during, and after the trial | P6 and PIS |
| Declaration of interests | 28 | Financial and other competing interests for principal investigators for the overall trial and each study site | 20 |
| Access to data | 29 | Statement of who will have access to the final trial dataset, and disclosure of contractual agreements that limit such access for investigators | See PIS |
| Ancillary and post-trial care | 30 | Provisions, if any, for ancillary and post-trial care, and for compensation to those who suffer harm from trial participation | See PIS |
| Dissemination policy | 31a | Plans for investigators and sponsor to communicate trial results to participants, healthcare professionals, the public, and other relevant groups (eg, via publication, reporting in results databases, or other data sharing arrangements), including any publication restrictions | See PIS |
|  | 31b | Authorship eligibility guidelines and any intended use of professional writers | See PIS |
|  | 31c | Plans, if any, for granting public access to the full protocol, participant-level dataset, and statistical code |  |
| Appendices |  |  |  |
| Informed consent materials | 32 | Model consent form and other related documentation given to participants and authorised surrogates | See Consent Form Appendix 2 |
| Biological specimens | 33 | Plans for collection, laboratory evaluation, and storage of biological specimens for genetic or molecular analysis in the current trial and for future use in ancillary studies, if applicable |  |

*It is strongly recommended that this checklist be read in conjunction with the SPIRIT 2013 Explanation & Elaboration for important clarification on the items. Amendments to the protocol should be tracked and dated. The SPIRIT checklist is copyrighted by the SPIRIT Group under the Creative Commons “[Attribution-NonCommercial-NoDerivs 3.0 Unported](http://www.creativecommons.org/licenses/by-nc-nd/3.0/)” license.

Appendix 1: Patient Information Sheet Version 2 07.11.14

**Participant Information Sheet**

Study Title: **Coping with chronic pain**

Name of Principal Investigator: **Ana Howarth**

We would like to invite you to take part in a research study. Before you decide to take part you need to understand why the research is being done and what it will involve. Please take time to read the following carefully and ask us if anything is not clear.

**Part 1** of this leaflet tells you the purpose of this study and what it involves.

**Part 2** gives you more detailed information about the study.

**Part 1: What is the purpose of the study?**

The main aim of the study is to explore the effects of a 15 minute audio recording in managing chronic pain. It is an experimental study, which does not involve any additional drugs or external treatment. A PhD student from St George’s, University of London will run the study under supervision as part of their degree.

**Why have I been invited?**

Because you have been attending an outpatient clinic, you live with a chronic pain and you are aged 18 years or over you may be invited to participate. We are aiming to recruit 90 patients.

**Do I have to take part?**

No, you can decide whether or not to take part. If you decide to participate, you will be given this information sheet and asked to sign a consent form, one copy of which you will keep. You are free to withdraw at any time and without giving a reason. Deciding at any time to withdraw will not affect your medical care

.

**What will happen to me if I take part?**

You will be asked to complete some questionnaires about yourself, and then we will download some audio recordings onto a device of your choice (i.e. smart phone or iPad) or if you do not have such a device, you will be given an MP3 player with the audios already downloaded. You will be asked to listen to the first 15 minute recording and the researcher will remain with you while you do this in case you have any questions. You will then be asked to repeat some of the questionnaires you filled out prior to the recording.

Following this, we will ask you to take home the audios to listen to at least three more times during the following week. We will give you some questionnaires to complete during the week. . We will go through the questionnaires with you to make sure everything is clear. Before leaving, we will ask you to briefly discuss if there might be any difficulties with using the audios at home and if so, how you might overcome them. The clinic visit should take under 45 minutes (approximately 20mins completing questionnaires, 15mins listening to the audio and 5mins discussing the questionnaires and about using the audio at home.

A researcher will call you a week later to check how you have been getting on with listening to the audio recordings. The researcher will also remind you to post back the questionnaires (in a pre-paid envelope). We will encourage you to continue using the audios for another three weeks.

Four weeks after your clinic visit, the researcher will call you for a final time to see how you have found using the audio and again to remind you to post back the questionnaires

**What are the possible disadvantages and risks of taking part?**

There are no expected disadvantages or risks involved in taking part in this study.

**What are the possible benefits of taking part?**

We cannot promise the study will help you, but what we learn may help to improve methods for helping people with cope with chronic pain. You will be welcome to keep the recordings if you find them helpful.

**What if there is a problem?**

Any complaint about the way you have been dealt with in this study will be addressed. Detailed information is given in part 2.

**Who is managing and funding the research?**

St George’s, University of London is managing and funding the research.

**Will my taking part in the study be kept confidential?**

Yes, we will follow ethical and legal practice and all information about you will be handled in confidence. Your consultant or physiotherapist may be informed of your participation in the study. The details are included in Part 2. ***If you are considering taking part, please read part 2 before making any decision.***

**Part 2**

**What will happen if I don’t want to carry on with this study?**

If you withdraw from the study we will destroy any information you have given us and it will not affect the standard of care you receive.

**What if there is a problem?**

If you have a concern about any aspect of this study, you can contact the Academic Supervisor of the study (Professor Michael Ussher, Population Health Research Institute, St George’s University of London, SW17 0RE, 0778 662 8572, mussher@sgul.ac.uk). The normal complaints mechanisms of St George’s, University of London are also available to you (contact the Patients Advice & Liaison Service on 020 8725 2453).

**Will my taking part in the study be kept confidential?**

All information collected about you during the course of the research will be kept strictly confidential, and will be stored in locked cabinets in the Population Health Research Institute, SGUL. It will be identifiable by code number and will be available by password only to members of the Research Team.

**What will happen to the results of the research study?**

The results of the study will be published in scientific journals. If you wish, we will send you a summary of the results, written in everyday language. You will not be identified in any way in any report or publication.

**Who has reviewed the study?**

Ethical approval for the study was given by the NRES Camden & Islington Research Ethics Committee (14/LO/1912)

If you are interested in participating in this research study **please contact:**

**ANA HOWARTH by email at** [**p1306542@sgul.ac.uk**](mailto:p1306542@sgul.ac.uk)

**or by telephone on 07946 377 990.**

THANK YOU

Appendix 2: Consent Form Version 1, 17.09.14

REC Reference Number: 14/LO/1912

Patient Identification Number for this study: **_______**

**CONSENT FORM**

Title: **Coping with chronic pain**

Name of Principal Investigator: Ana Howarth

**Please initial box**

1. I confirm that I have read and understand the information sheet dated 17.09.14 (Version 2) for the above study and have had the opportunity to consider the information, ask questions and have these answered satisfactorily.
2. I understand that my participation is voluntary and that I am free to withdraw at any time, without giving any reason and without my medical care or legal rights being affected.
3. I understand that relevant sections of my medical notes and data collected during the study may be looked at by responsible individuals from St George’s University of London (SGUL) and/or St George’s NHS Healthcare Trust (SGHT) or from regulatory authorities where it is relevant to my taking part in research. I give permission for these individuals to have access to my medical notes and data.
4. I agree to my consultant or physiotherapist being informed of my participation in the study.
5. I agree to take part in the above study.

________________________ ___________ ___________________

Name of Patient Date Signature

_________________________ ___________ ___________________

Name of person taking consent Date Signature

***When completed 1 copy to be given to patient, 1 to be placed with study documentation, 1 to be placed with patient’s medical***
