# Supplementary material for: Pilot study evaluating a brief mindfulness intervention for those with chronic pain: study protocol for a randomized controlled trial
Source: Trials. 2016 Jun 2;17:273. doi: 10.1186/s13063-016-1405-2 (PMC4890280; doi:10.1186/s13063-016-1405-2)
Supplement: Additional file 2: — SPIRIT figure. (DOC 52 kb) [file 13063_2016_1405_MOESM2_ESM.doc]

Figure 2. Schedule of enrolment, interventions, and assessments.

| **STUDY PERIOD** | | | | | | | |
| --- | --- | --- | --- | --- | --- | --- | --- |
|  | | | **Post-allocation** | | | | |
| **TIME POINT:** | **Enrolment** | **Allocation** | **Baseline** | **During week 1** | **At one week** | **During month** | **At one month** |
| **Eligibility screen** | X |  |  |  |  |  |  |
| **Informed consent** | X |  |  |  |  |  |  |
| **Baseline measures (measures listed below under assessments)** | X |  |  |  |  |  |  |
| **Allocation** |  | X |  |  |  |  |  |
| **INTERVENTIONS:** | | | | | | | |
| **Treatment Group** |  |  | ◆**----------------------------------------------------------------**◆ | | | | |
| **Control Group** |  |  | ◆**----------------------------------------------------------------**◆ | | | | |
| **ASSESSMENTS:** | | | | | | | |
| **Background and pain related questionnaire** |  |  | X |  |  |  |  |
| **Pain self-efficacy item** |  |  | X |  |  |  | X |
| **Pain and physical function item** |  |  | X |  |  |  | X |
| **Mood questionnaire (HADS*)** |  |  | X |  |  |  |  |
| **Mindfulness questionnaire (CAMS-R*)** |  |  | X |  | X |  | X |
| **Pain specific questionnaire (BPI*)** |  |  | X |  |  |  |  |
| **Pain catastrophizing questionnaire (PCS*)** |  |  | X |  |  |  | X |
| **HRQoL questionnaire**  **(EQ-5D-5L*)** |  |  | X |  |  |  | X |
| **Brief psychological measures (before and after intervention)** |  |  | X | X |  |  |  |
| **Experience of audio items** |  |  |  |  | X |  |  |
| **Previous experience** |  |  |  |  | X |  |  |
| **Self-monitoring Table** |  |  |  | X |  | X |  |

*HADS (Hospital Anxiety and Depression Scale), CAMS-R (Cognitive and Affective Mindfulness Scale Revised), BPI (Brief Pain Inventory), PCS (Pain Catastrophizing Scale), EQ-5D-5L (EuroQuol - 5 Dimensions - 5 Levels)
